# A Model Document

**Published:** 1869-11

**Authors:** 


A Model Document.—The following is a literal transcript of a document written by a practicing physician in this city, and offered as evidence in a case in the Police Court recently. If the writer's knowledge of medicine is as extended as his orthographical acquirements, the profession would do well to hunt him out:

" I was called on to go and see Samel snapinsca on the 11 september i found him in bead and was brused very bad his ribs on the left side was varey badly brused and the left lung apeers to be ingered from the bruses and there was severil bruses on his body besides."—*Detroit Free Press.*

